# Temperate Climate Niche for *Cryptococcus gattii* in Northern Europe

**DOI:** 10.3201/eid1801.111190

**Published:** 2012-01

**Authors:** Anuradha Chowdhary, Harbans S. Randhawa, Teun Boekhout, Ferry Hagen, Corné H. Klaassen, Jacques F. Meis

**Affiliations:** University of Delhi, Delhi, India (A. Chowdhary, H.S. Randhawa);; Centraalbureau voor Schimmelcultures Fungal Biodiversity Center, Utrecht, the Netherlands (T. Boekhout);; Canisius Wilhelmina Hospital, Nijmegen, the Netherlands (F. Hagen, C.H. Klaassen, J.F. Meis);; Radboud University Nijmegen Medical Centre, Nijmegen (J.F. Meis)

**Keywords:** Cryptococcus gattii, the Netherlands, Europe, fungi, climate

**To the Editor**: *Cryptococcus gattii* was considered to be geographically restricted to countries with tropical and subtropical climates until 1999, when an outbreak of cryptococcosis in humans and animals occurred in the temperate climate of Vancouver Island, British Columbia, Canada (*1*). Montagna et al. reported the first environmental *C. gattii* in Europe from the Mediterranean region of Italy; these authors isolated it from 11 (4.3%) of 255 samples of plant detritus of *Eucalyptus camaldulensis* trees collected from the residential locality of an autochthonous case of cryptococcal meningitis caused by *C. gattii* in Apulia (*2*). These observations were recently substantiated by the isolation of *C. gattii* from plant debris of trees belonging to *Ceratonia siliqua* (carob), *Pinus halepensis* (stone pine), and *E. camaldulensis* in Spain (*3*). We report environmental isolation of the primary pathogenic fungus *C. gattii* from a forest in Berg en Dal, the Netherlands, which extends its geographic distribution to the temperate climate of northern Europe.

We investigated 112 decayed wood samples collected from inside trunk hollows of 52 living trees belonging to 5 families during April–May 2011 in Nijmegen, the Netherlands. The trees sampled were chestnut (*Castanea sativa*, n = 24), Douglas fir (*Pseudotsuga menziesii*, n = 17), oak (*Quercus macranthera*, n = 6), walnut (*Juglans regia*, n = 3), and mulberry (*Morus alba*, n = 2). The main criterion in selecting a tree for sampling was advanced age and presence of large trunk hollows variably sheltered from sunlight. The sampled sites had no bird nests and were apparently free from avian excreta. The decayed wood samples were collected with an in-house swabbing technique by using simplified Staib niger seed agar as described (*4*). The plates were incubated at 30°C and periodically observed up to 7 days for isolation of *C. gattii* and *C. neoformans*. Suspected colonies of *Cryptococcus* spp. were purified by dilution plating and identified by their morphologic and biochemical profiles, including development of blue color on l-canavanine-glycine bromothymol blue medium.

Identity of the isolates was confirmed by sequencing the internal transcriped spacer and D1/D2 regions, and they were genotyped by using amplified fragment-length polymorphism (AFLP) fingerprinting and multilocus sequence typing (MLST). The MLST loci *CAP10*, *CAP59*, *GPD1*, IGS, *LAC1*, *MPD1*, *PLB1*, *SOD1*, *TEF1*α, and *URA5* of the environmental *C. gattii* isolates were amplified and sequenced, and data were compared with MLST data from a large *C. gattii* population study (*5*) and with a recently published set of clinical, animal, and environmental *C. gattii* isolates from Mediterranean Europe and the Netherlands (Figure) (*3**,**6**,**7*). In addition, the mating type was determined with PCR by using mating type–specific primers for the *STE12*a and α alleles (*8*).

**Figure:**
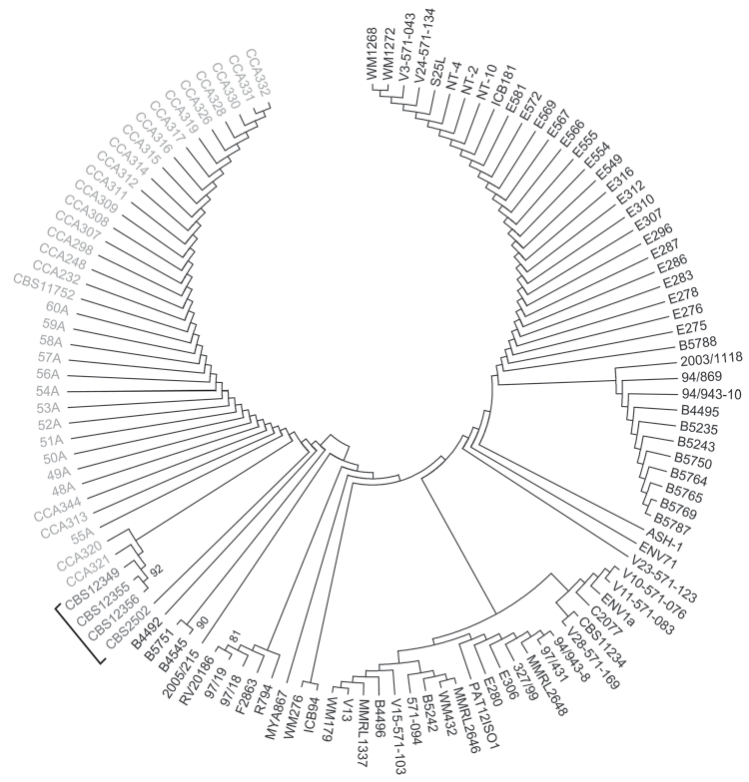
Unrooted bootstrap maximum-likelihood phylogenetic multilocus sequence typing analysis of *Cryptococcus gattii* genotype AFLP4/VGI isolates based on 7 unlinked nuclear loci (*5*). Red indicates the 3 *C. gattii* isolates from the Netherlands cultured from Douglas fir (*Pseudotsuga menziesii*) (CBS12349, CBS12355, CBS12356) and 1 clinical isolate from 1957 from the Netherlands (CBS2502) (*6*). Green indicates the previously observed European Mediterranean cluster, with clinical, animal and environmental isolates (*3*). All other *C. gattii* AFLP4/VGI isolates originate mainly from Australia, Africa, and South America, as described (*5*). The isolates from the Netherlands are closely related to isolates that originated from the Mediterranean region. Numbers next to branches show bootstrap support (>80).

Four strains of *C. neoformans* species complex were isolated from the 112 decayed wood samples examined from 52 trees. One strain that originated from an oak tree (*Q. macranthera*), was identified as *C. neoformans* var. *grubii*. The remaining 3 strains, all originating from different hollows in a Douglas fir tree, were identified as *C. gattii* genotype AFLP4/VGI and mating type α. The strains were deposited at the CBS-KNAW (Centraalbureau voor Schimmelcultures/Royal Netherlands Academy of Arts and Sciences) Fungal Biodiversity Centre (accession nos. CBS12349, CBS12355, and 12356), and the sequences were deposited in GenBank (accession nos. JN982044–JN982073).

MLST analysis showed that the *C. gattii* isolates in our study are more closely related to the clinical isolate from the Netherlands (*6*) and to the clinical and environmental *C. gattii* isolates (AFLP4/VGI) reported from the Netherlands and other countries in Europe than to isolates from outside Europe (*3**,**7**,**8*). The autochthonous *C. gattii* AFLP4/VGI isolate, CBS2502 (earlier identified as *C. neoformans*) isolate from the Netherlands was recovered postmortem in 1957 from the lungs of a pregnant woman with cryptococcosis (*6*). This patient came from a low socioeconomic strata, was unlikely to have traveled outside the Netherlands, and probably acquired the infection indigenously from an environmental source (*6*).

Furthermore, genotype AFLP4/VGI appears to be the genotype of *C. gattii* prevalent in Europe (*3**,**7**,**8*). Outside Europe, *C. gattii* has been reported from Douglas fir trees in Vancouver Island, British Columbia, Canada; however, those isolates represented another molecular type, i.e., AFLP6/VGII (*9*). Genotype AFLP 4/VGI *C. gattii* isolates have been implicated in human infections in that region, but to our knowledge, no environmental isolates have been found until now.

Our detection of *C. gattii* in the environment and its previous isolation from a clinical case in the Netherlands suggests that this pathogen is endemic to the temperate climate of northern Europe. This suggestion agrees with the concept emerging from a decade of investigations in Canada and the Pacific Northwest that the geographic distribution of *C. gattii* extends to the temperate region, albeit with another AFLP genotype (*1**,**9**,**10*). Further environmental studies are likely to show a wider spectrum of host trees and higher environmental prevalence of *C. gattii* in this continent than what appears in the literature.
